# The effects of linagliptin on endothelial function and global arginine bioavailability ratio in coronary artery disease patients with early diabetes: study protocol for a randomized controlled trial

**DOI:** 10.1186/s13063-016-1627-3

**Published:** 2016-10-13

**Authors:** Norbert J. Tripolt, Felix Aberer, Regina Riedl, Barbara Hutz, Jasmin Url, Gudrun Dimsity, Andreas Meinitzer, Tatjana Stojakovic, Ronald Hödl, Marianne Brodmann, Franz Hafner, Harald Sourij

**Affiliations:** 1 Department of Internal Medicine, Division of Endocrinology and Diabetology, Cardiovascular Diabetology Research Group, Medical University of Graz, Graz, Austria; 2 Department of Internal Medicine, Division of Angiology, Medical University of Graz, Graz, Austria; 3 Medical University of Graz, Institute for Medical Informatics, Statistics and Documentation, Graz, Austria; 4 Clinical Institute of Medical and Chemical Laboratory Diagnostics, Graz, Austria; 5 Center for Cardiovascular Rehabilitation, St. Radegund, Austria; 6 Center for Biomarker Research in Medicine, CBmed, Graz, Austria

**Keywords:** Linagliptin, DPP-4 inhibitor, Endothelial function, Global arginine bioavailability ratio, Coronary artery disease, Type 2 diabetes mellitus

## Abstract

**Background:**

Patients with type 2 diabetes (T2DM) are at increased risk for macrovascular events as well as for microvascular complications. There is evidence that in patients with coronary artery disease (CAD), about 35 % suffer from manifest T2DM. Early glucose-lowering intervention in subjects with T2DM has been demonstrated to be beneficial in terms of cardiovascular risk reduction. But thus far, no data are available regarding investigating the impact of linagliptin treatment in patients with early diabetes on cardiovascular endpoints or surrogate parameters. Therefore, the aim of this study is to investigate the effects of linagliptin in CAD patients with early T2DM on various cardiovascular surrogate measurements including mechanical and biochemical endothelial function assessments.

**Methods/design:**

Forty-two subjects with early diabetes and CAD are included in this investigator-driven, randomized, placebo-controlled, double-blind, phase IV, single-center study. Participants will be randomized to receive either linagliptin (5 mg) administered once daily per os or placebo for 12 weeks. Before and after the intervention, evaluation of endothelial function (flow-mediated dilatation and biochemical biomarkers) and a Meal Tolerance Test are performed.

**Discussion:**

Cardiovascular surrogate parameters, such as endothelial function, are able to provide insights into the potential mechanisms of the cardiovascular effects of antihyperglycemic agents. Currently ongoing trials do not specifically focus on early diabetes as a target of intervention and we therefore believe that our study will contribute to a better understanding of the cardiovascular effects of dipeptidylpeptidase-4 (DPP-4) inhibitors in early diabetes.

**Trial registration:**

NCT02350478. Registered 26 January 2015. Protocol date/version 24 October 2014/version 2.4

EudraCT number: 2013-000330-35

**Electronic supplementary material:**

The online version of this article (doi:10.1186/s13063-016-1627-3) contains supplementary material, which is available to authorized users.

## Background

Patients with type 2 diabetes (T2DM) are at increased risk of macrovascular events as well as of microvascular complications [1]. It is well-known that the pathophysiologic process of T2DM starts many years before the diagnosis can be made on the basis of elevated fasting blood glucose or pathologic glucose tolerance evaluation [2], and in particular the data from the United Kingdom Prospective Diabetes Study (UKPDS) and the UKPDS post-trial monitoring highlighted the importance of an early glucose-lowering intervention in patients with T2DM for the reduction of future microvascular and macrovascular complications [3, 4]. We [5, 6], and in particular the Euro Heart Survey on Diabetes and the Heart [7], have demonstrated that disturbances of glucose metabolism are present in approximately two thirds of patients with CAD, and current joint guidelines of the European Society of Cardiology (ESC) and the European Association for the Study of Diabetes (EASD) recommend screening for dysglycemic states in this patient population [8].

Dipeptidylpeptidase-4 (DPP-4) inhibitors increase endogenous glucagon-like peptide-1 (GLP-1) levels and GLP-1 in turn increases insulin release from pancreatic beta cells in a glucose-dependent manner as well as suppressing glucagon secretion from pancreatic alpha cells. Investigations in T2DM patients have shown that this drug class lowers both fasting and postchallenge or postmeal glucose levels, and hence glycosylated hemoglobin (HbA1c), and is well-tolerated.

However, the lowering of the surrogate measurement HbA1c level has not necessarily turned out to translate into a reduced number of cardiovascular events in patients with T2DM and, therefore, the American Food and Drug Administration (FDA) [9] and the EMA (European Medicines Agency) [10] issued in 2008 and 2010, respectively, guidance for new glucose-lowering drugs, requiring at least the proof of cardiovascular safety. Three cardiovascular outcome trials with the DPP-4 inhibitors alogliptin, saxagliptin and sitagliptin have been published to date [11–13] and have demonstrated cardiovascular safety albeit not cardiovascular superiority to usual diabetes care without DPP-4 inhibitors. Two outcome trials with linagliptin (CAROLINA [14] and CARMELINA - NCT01897532) are underway, of which the former is performed in subjects with early diabetes.

A major limitation of the published studies is that they included primarily patients with longstanding diabetes (median duration of between 7.1 and 11.6 years in EXAMINE, SAVOR-TIMI53 and TECOS) while data from the UKPDS suggest that patients benefit most from an early glucose-lowering intervention rather than a later intervention in the course of the disease. The cardiovascular impact of DPP-4 inhibitors in early treatment remains unclear and there is currently no ongoing trial in this patient population given the low event rate and the need for long-term follow-up.

A well-known and validated cardiovascular surrogate parameter for future cardiovascular events is endothelial dysfunction. We and others have shown previously that endothelial dysfunction is present in patients with CAD and early diabetes and can be improved by pharmacologic intervention [15, 16]. Data on the effect of DPP-4 inhibitors on endothelial function are sparse and the results of these trials are conflicting. While Nakamura et al. [17] demonstrated an improvement of endothelial function with sitagliptin, Ayaori and coworkers [18] showed a worsening of endothelial function with the same drug. However, none of these trials was performed in a placebo-controlled fashion.

Therefore, we aim to perform a randomized, placebo-controlled, double-blind trial investigating the effect of the DPP-4 inhibitor linagliptin on endothelial function and further biochemical markers of vascular function, which will help to better understand the cardiovascular effects of DPP-4 inhibitors in early diabetes.

### Aim of the study

The aim of our current study is to investigate the effects of linagliptin in CAD patients with early T2DM on various cardiovascular surrogate measurements including mechanical and biochemical endothelial function assessments.

## Methods/design

### Design

**Fig. 1:**
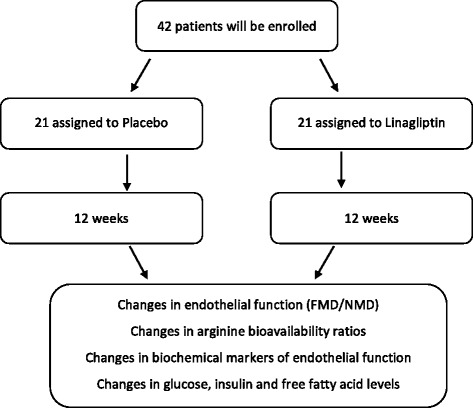
Flow diagram of the study design (*study dropouts will be replaced)

This is an investigator-driven, prospective, randomized, placebo-controlled, double-blind, phase IV, single-center study to evaluate the effect of linagliptin 5 mg once daily on cardiovascular surrogate measures in patients with early diabetes and CAD (Fig. 1). The trial was approved by the Ethics Committee of the Medical University of Graz (25-295 ex 12/13) and is conducted at the Division of Endocrinology and Diabetology at the Medical University of Graz (academic hospital), Austria. All participants are asked to provide written informed consent before entering the study. Clinical trial authorization has been obtained from the Austrian Agency for Health and Food Safety (AGES) (EudraCT number: 2013-000330-35). Important protocol modifications will be reported to the local Ethics Committee of the Medical University of Graz and, if necessary, to the AGES. This study follows the international recommendations for interventional trials (see the Standard Protocol Items: Recommendations for Interventional Trials (SPIRIT) checklist in Additional file 1).

### Inclusion and exclusion criteria

Participants for the trial are aged 40 to 80 years with early T2DM (i.e., 2-h glucose post oral glucose exposure (75 mg) > 200 mg/dl or T2DM treated with diet only or on a stable dose of metformin monotherapy), a recent glycosylated hemoglobin (HbA1c) level of between 6.0 (42 mmol/mol) and 9.0 % (75 mmol/mol) and coronary atherosclerosis (diagnosed either by coronary angiography or coronary computer tomography).

The exclusion criteria are: acute coronary syndrome or a cerebrovascular event within the previous 4 weeks; Body Mass Index (BMI) > 35.0 kg/m^2^; serum creatinine level > 2.5 mg/dl; aspartate aminotransferase (AST)/alanine aminotransferase (ALT) more than threefold the upper limit of normal; heart failure more severe than NYHA class II; uncontrolled hypertension (blood pressure above 165/100 mmHg); treatment with orally administered steroids; use of a new-onset statin or angiotensin converting enzyme (ACE) inhibitor within the previous 6 weeks; known malignancy; known intolerance to DPP-4 inhibitors; any antidiabetic therapy except metformin; and pregnant or breastfeeding women. We amended the reduction of the lower boundary of the range of HbA1c level from 6.5 (48 mmol/mol) to 6.0 % (42 mmol/mol) after the enrollment of 10 subjects to increase the recruitment rate.

### Primary objective

The primary objective of this trial is to determine whether 12-week linagliptin treatment is associated with an improvement in endothelial function in patients with early diabetes.

### Secondary objective

1. To investigate the effect of linagliptin treatment on arginine bioavailability ratios (global arginine bioavailability ratio (GABR) and arginine to ornithine ratio)
2. To study the impact of linagliptin treatment on biochemical markers of endothelial function (soluble vascular cell adhesion molecule-1 (sVCAM-1) and soluble intercellular adhesion molecule-1 (sICAM-1)
3. To evaluate the Area Under Curve (AUC) of glucose, insulin and free fatty acids (FFA) during the Meal Tolerance Test (MTT)

### Outcome measurements

The primary outcome is the difference in endothelial function measured by FMD (flow-mediated dilatation) before and after 12 weeks of treatment with linagliptin or matched placebo.

The secondary outcome measures are GABR, arginine to ornithine ratio, sVCAM-1 level, sICAM-1 level and AUC of glucose, insulin and FFA during the MTT.

### Interventions

The subjects receive either linagliptin 5 mg (licensed dose for treatment of T2DM) or matched placebo tables administered per os once daily for 12 weeks. The drug and placebo are supplied by Boehringer Ingelheim as bulkware study medication. The study capsules, linagliptin and placebo are identical in appearance and all are tasteless and colorless. The pharmacy at the Medical University of Graz, Austria packs the medication as 12-week supplies for study participants and labels the study medication according to current regulatory requirements.

Patients are asked not to adjust their antidiabetic treatment during the study period. However, if glycemic control appears to be inappropriately high during these 12 weeks, additional glucose-lowering drugs may be used at the discretion of the principal investigator. Unless contraindicated, the first choice for rescue medication should be a sulfonylurea.

The subjects are instructed to return all unused or partly-used medication and packaging from medication at the follow-up visit. Tablets are counted at the end of the 12 weeks of the treatment period to verify medication adherence. Significant nonadherence is defined as missing 20 % or more doses of trial medication during the 12 weeks of the treatment period. In the case of study medication noncompliance, participants would continue to be followed to their last visit. These data would not be part of the per-protocol analysis but would form part of an intention-to-treat (ITT) analysis.

Withdrawal of consent and major protocol violations will lead to an early study termination for the subject

### Eligibility and recruitment

The study population will consist of 48 subjects with early diabetes and CAD. Subjects are identified from the outpatient clinic at the Department of Endocrinology and Diabetology, the Division of Cardiology, via primary care and advertisements. The clinic staff informs patients about the possibility of being enrolled in this study. No study-related procedures are undertaken before obtaining informed consent. Then, the study team explains the study procedures in detail and asks the participant about their willingness to participate in this research study. After informed consent is signed and obtained, participants are given a signed copy of the Informed Consent Document and are assigned a screening identification number. Any use of study samples that is outside the scope of the objectives of this protocol will be submitted for prior review and approval by the appropriate Institutional Review Board.

### Screening

Screening of participants consists of the evaluation of inclusion and exclusion criteria. After informed consent is obtained, a numbered subject folder is created for potential participants, including information about medical history, physical examination and their actual medication. Glycosylated hemoglobin (HbA1c), serum creatinine and AST/ALT levels obtained from venous blood are checked by laboratory investigation. Women of reproductive potential undergo urine pregnancy testing. Eligible subjects are scheduled for the following study visits (Table 1).

**Table 1:**
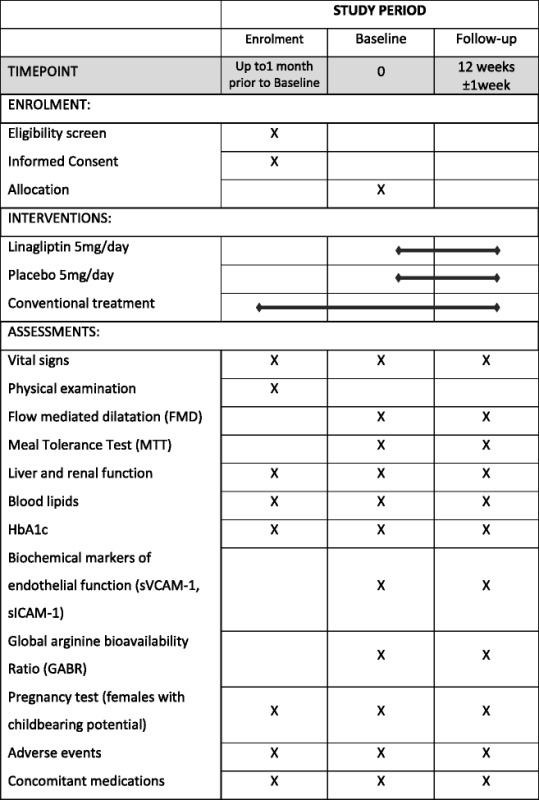
Schedule of enrollment, interventions and assessments

*sVCAM-1* Soluble Vascular Cell Adhesion Molecule-1, *sICAM-1* soluble Intercellular Adhesion Molecule-1

### Randomization and blinding

Participants are randomized to one of the two study arms (intervention versus placebo) in the ratio 1:1 in real time by using the web-based randomization tool “Randomizer for Clinical Trials” (https://www.randomizer.at/). The software’s compliance with Good Clinical Practice (GCP) has been confirmed by the Austrian Agency for Health and Food Safety (AGES). The randomization plan is designed by the Institute of Medical Informatics, Statistics and Documentation, Medical University Graz, Austria, which passes the treatment codes to the Pharmacy of the University Hospital Graz for adequate labeling and blinding of the medication. The labels of the study medication display the name of the trial, the name of the investigational medicinal product and contact details of the principal investigator. Subject numbers are assigned sequentially as each subject enters the study. The study is subject- and investigator-blinded during the treatment periods from visit 2 until the end of the study. In the rare case of a requirement to unblind study medication, the principal investigator needs to give permission for unblinding. The unblinding list is held by the Institute of Medical Informatics, Statistics and Documentation, Medical University of Graz, which is not involved in study investigations.

### Reporting procedure for all adverse events

All adverse events (AE), serious (SAE) and nonserious, are collected, documented and reported on the appropriate Case Report Forms (CRFs)/SAE Reporting Forms once informed consent has been signed and this will end 28 days after completing the trial. The following information is recorded:

- ▪ Participant details
- ▪ Adverse event description
- ▪ Start date of event
- ▪ End date of event
- ▪ Outcome of event
- ▪ Severity of event
- ▪ Treatment required
- ▪ Relationship to study drug (i.e., causality/relatedness)
- ▪ Assessment of relatedness to other suspect drug or device
- ▪ Action taken with study drug
- ▪ Whether subject withdrawn due to an adverse event
- ▪ Whether the event is serious
- ▪ Follow-up information will be recorded as necessary

AEs considered to be related to study medication as judged by a medically qualified investigator are followed until resolution or the event is considered stable. All related AEs that result in a subject’s withdrawal from the study or are present at the end of the study are followed up until a satisfactory resolution occurs.

### Reporting procedure for serious adverse events

The investigator reports any SAEs and nonserious AEs which are relevant for reported SAEs and AEs of Special Interest to Boehringer Ingelheim, Pharmacovigilance Unique Entry Point, which will subsequently report the SAE to the Ethics Committee and local authorities.

### Physical measurements

Anthropometric measurements are performed on each participant at each study visit. Weight is measured with the patient standing and then registered after rounding it to the nearest 500 grams. Height is measured using a metric tape with the patient standing against the wall, and the value marked by a ruler placed horizontally on the vertex of the patient’s head. BMI is estimated using the weight in kilograms divided by the second power of the height expressed in meters. Blood pressure is taken using an automated sphygmomanometer Boso Medicus Uno (Bosch & Sohn GmbH, Juningen, Germany) after a 5-min rest in sedentary body position. Number of beats per min is measured from the radial artery by manual palpation.

### Measurement of flow-mediated dilatation (FMD) of the brachial artery

Endothelium-dependent FMD following reactive hyperemia and endothelium-independent nitroglycerin-mediated dilatation (NMD) following administration of glyceryl trinitrate (GTN) are examined in the brachial artery by a trained physician according to the guidelines described by Coretti et al. [19]. Vasodilatation of the right brachial artery is measured using a 9–14 MHz, linear array, high-resolution transducer (ACUSON S2000 ultrasound system, SIEMENS Healthcare, 91052 Erlangen, Germany). After a 5-min rest in the supine position the brachial artery is examined in a longitudinal plane above the antecubital fossa by continuous grayscale imaging in a segment with definitive intimal interfaces between the lumen and the vascular wall. The distance between the two intimal interfaces is measured at least three times in the end-diastolic phase using echocardiographic (ECG). A blood pressure cuff is placed on the forearm and inflated to a pressure of 250 mmHg for 5 min. The postischemic diameter of the brachial artery is taken 45 s after cuff release. At least 15 min later, the diameter between the two intimal lines is recorded immediately before and 3 min after sublingual administration of 0.4 mg GTN. FMD diameter is calculated as the average of the three diameter measurements following reactive hyperemia. FMD and NMD are calculated as the percentage change in diameter compared to baseline.

### Meal Tolerance Test (MTT)

The MTT is performed after an overnight fast (apart from water). A standard-gauge cannula is placed into a subcutaneous vein for blood sampling. In order to prevent blood clotting in the cannula and to keep the cannula working it will be occasionally flushed with sterile normal saline. Study medication will be given (time −15 min) before the meal. A pre-meal blood sample will be taken (−5 min) and then all subjects are asked to drink Fortimel compact (10 kcal/kg) over a period of 2 − 4 min (time 0 min). During the meal test further blood samples will be taken at 15, 30, 60 and 120 min. All samples are used for determination of glucose, insulin and FFA levels. The blood at each time point is placed into a fluoride oxalate tube (1 ml) for plasma glucose and into a serum tube for insulin and FFA.

### Laboratory measurements

Insulin and c-peptide will be measured by chemiluminescence on an ADVIA Centaur system (Siemens Healthcare Diagnostics, Eschborn, Germany). Lipoproteins will be separated using a combined ultracentrifugation-precipitation method (β-quantification). Cholesterol, triglyceride and phospholipid levels will be determined enzymatically (DiaSys, Holzheim, Germany). Apolipoprotein (apo) AI, apo B and lipoprotein(a) levels will be measured by immunoturbidimetry (Greiner, Flacht, Germany). Lipoprotein analyses will be performed on an Olympus AU640 analyzer (Olympus Diagnostika, Hamburg, Germany). Free fatty acids (FFA) will be measured enzymatically (Wako Chemical, Neuss, Germany). Routine parameters will be determined using a cobas® analyzer (Roche Diagnostics, Mannheim, Germany). Inflammatory markers, such as sVCAM-1 and sICAM-1, will be measured by enzyme-linked immunosorbent assays.

### Arginine bioavailability ratios

L-arginine, L-citrulline and L-ornithine will be measured using modifications of previously described chromatographic methods [20, 21]. Briefly, after precipitation of ethylenediaminetetraacetic acid (EDTA) plasma with perchloric acid following neutralization of the supernatant with sodium carbonate, the extracted amino acids are derivatized with o-phthalaldehyde and separated on a reversed phase column with gradient elution. Quantification will be performed using ratios of the fluorescence signals of the amino acids of interest to the internal standard norvaline in comparison to the appropriated calibration curves. Intra-assay and interassay CVs (coefficient of variation) are all below 10 %. The global arginine bioavailability ratio (GABR) will be calculated from L-arginine divided by the sum of (L-ornithine plus L-citrulline). The L-arginine to L-ornithine ratio will be calculated by dividing the L-arginine by the L-ornithine levels.

## Statistical considerations

A detailed Statistical Analysis Plan (SAP) will be prepared. Before unblinding and locking the data for statistical analysis, a review of all data will take place and the SAP will be finalized and approved. Should the SAP and this protocol differ, the methods in the SAP will prevail.

### Definition of analysis populations

Study participants who withdraw their informed consent are excluded from the study and will not be included in any statistical analysis.

The analysis populations are defined as follows:

The ITT population will include all randomized patients with a recorded primary outcome measure (i.e., the baseline and follow-up FMD measurement). All patients will be analyzed according to the randomized treatment group. The primary analysis will be performed on the ITT population.

The per-protocol population will consist of all patients with recorded baseline and follow-up FMD measurements and study medication compliance > 80 %. Noncompliance is defined as missing 20 % or more doses of trial medication during the 12-week treatment period.

The safety population will include all patients who received at least one dose of the study medication. The study participants will be analyzed according to the treatment received.

### General aspects

The analysis of the collected data in the study will be performed using SAS v9.4. Continuous variables will be presented as means, standard deviation (SD), median and minimum and maximum; for categorical data, frequencies and relative frequencies will be used.

### Demographic and baseline characteristics

The demographic and baseline characteristics will be summarized and compared between the groups descriptively.

### Analysis of the primary outcome

The primary outcome, the change in endothelial function measured by FMD at baseline and after 12 weeks of treatment with linagliptin or placebo will be analyzed using analysis of covariance (ANCOVA) with FMD at week 12 as the dependent variable, and the baseline FMD and treatment group as covariates. A two-sided *p* value of < 0.05 is considered to indicate statistical significance.

### Analysis of the secondary outcomes

For the secondary outcomes, arginine bioavailability ratios (GABR, arginine to ornithine ratio), biochemical markers of endothelial function (sVCAM-1, sICAM-1) and the evaluation of AUC of glucose, insulin and FFA during the MTT, group comparisons will be performed using parametric or nonparametric methods for unpaired data (as appropriate). Comparisons within groups will be performed using a paired *t* test or the Wilcoxon rank-sum test.

### Analysis of the safety parameters

Safety parameters will be summarized as interval or categorical summaries as appropriate. Listings and summary tables of AEs will be presented by treatment group and relationship, seriousness and intensity.

### Missing data handling

All available data will be used in the analysis and data summaries. There will be no imputation of any missing data. The reason that data are missing will be documented and reported.

### Sample size

The sample size was estimated using the free calculator available from http://www.quantitativeskills.com/sisa/calculations/samsize.htm. For the sample size estimation we assumed a relative 30 % improvement of endothelial function in the linagliptin group and no changes from baseline to week 12 in the placebo group. Although effect sizes and SDs vary substantially, we based our estimation on a baseline FMD of approximately 5.0 % and a SD of 1.5 % (absolute % FMD) [22] a total of 42 subjects will be needed to detect this difference with a two-sided alpha of 0.05 and a power of 0.90. For every subject dropping out during the study (withdrawal of consent, lost to follow-up) an additional subject will be enrolled to make sure the trial is adequately powered. To consider a dropout rate of approximately 15–20 %, we will randomize a maximum of 50 subjects.

## Data collection

### Electronic Case Report Form (eCRF)

Study data will be captured on a web-based open source eCRF (OpenClinica).

Characteristics at baseline and follow-up visits are gathered: age, sex, weight, height, BMI, waist and hip circumference, blood pressure, medical history, smoking behavior, medication, FMD and MTT results. Privacy of the patients is guaranteed; stored data and materials will only be identifiable to the person by a sequentially assigned subject number. The eCRF is designed in accordance with the requirements of the study protocol and complies with regulatory requirements. Access to the eCRF is password-protected and the password is only given to site personnel. Data generated throughout the study are monitored, and the eCRFs are checked against the subject records for accuracy and accuracy. Following completion of the eCRFs, the data are checked electronically for consistency and plausibility by predefined range checks. If necessary, automatic queries are generated for questionable data.

### Data quality control

The research assistant and the investigators meet monthly to review the study progress and procedures and to discuss any AEs or dropouts. In view of the small sample size, short study period and phase of the trial, a Data Monitoring Committee, interim analysis and stopping guidelines were not including in the trial design. The data collection, management, analysis, interpretation and production of publications is independent of the funding bodies and other competing interests. The trial results will be disseminated via journal publication and conference presentation without exposing the identity of the trial subjects.

## Monitoring

Monitoring is undertaken according to ICH Good Clinical Practice guidelines and the study monitoring plan. The study monitor is suitably trained, qualified and experienced to perform this task. Data are evaluated for compliance with the protocol and accuracy in relation to source documents. The following data are assessed:

- ▪ Written informed consent
- ▪ Flow chart filled in for included and excluded subjects
- ▪ Trial progress
- ▪ Primary and secondary outcome collection
- ▪ Severe adverse events
- ▪ Drug accountability of the study treatment

## Discussion

A great variety of antidiabetic agents are available for T2DM treatment. These treatments differ in their mechanisms of drug action and their long-term outcomes. The FDA and the EMA require cardiovascular trials for all new antihyperglycemic drugs in order to prove the safety of these agents. Cardiovascular surrogate parameters, such as endothelial function, cannot replace outcome trials but are able to provide insights into the potential mechanisms of the cardiovascular effects of antihyperglycemic agents. Moreover, currently ongoing trials do not specifically focus on early diabetes as a target of intervention and we therefore believe that our study will contribute to a better understanding of cardiovascular effects of DPP-4 inhibitors in early diabetes.

### Trial status

The trial is currently enrolling patients. As of 28 February 2016, 80 % of the patients have been enrolled in the study.

## Additional file

Additional file 1: SPIRIT checklist. (PDF 74 kb)

## Abbreviations

ACE: Angiotensin converting enzyme
AE: Adverse event
AGES: Austrian Agency for Health and Food Safety
ALT: Alanine transaminase
ANCOVA: Analysis of covariance
AST: Aspartate transaminase
AUC: Area Under Curve
BMI: Body Mass Index
CAD: Coronary artery disease
CV: Coefficient of variance
DPP-4: Dipeptidylpeptidase-4
ECG: echocardiography
eCRF: Electronic Case Report Form
EDTA: Ethylenediaminetetraacetate
EMEA: European Medicines Agency
FDA: Food and Drug Administration
FFA: Free fatty acids
FMD: Flow-mediated dilatation
GABR: Global arginine bioavailability ratio
GLP-1: Glucagon-like-peptide-1
GTN: Glyceryl trinitrate
HbA1c: Glycosylated haemoglobin
ITT: Intention-to-treat
MTT: Meal Tolerance Test
NMD: Nitroglycerin-mediated dilatation
SAE: Serious adverse event
SAP: Statistical Analysis Plan
SD: standard deviation
sICAM-1: soluble intercellular adhesion molecule-1
SUSAR: Suspected unexpected serious adverse reaction
sVCAM-1: Soluble vascular cell adhesion molecule-1
T2DM: Type 2 diabetes mellitus
UKPDS: United Kingdom Prospective Diabetes Study
